# Opportunities within the meat supply chain in Africa—The case of beef production in Northern Ghana

**DOI:** 10.1371/journal.pone.0260668

**Published:** 2022-01-18

**Authors:** Áron Vaskó, Imre Vida, László Vasa, Frederick Adzitey

**Affiliations:** 1 University for Development Studies, Tamale, Ghana; 2 Hungarian University of Agriculture and Life Sciences, Gödöllő, Hungary; 3 Institute for Foreign Affairs and Trade, Széchenyi István University, Győr, Hungary; Wroclaw University of Economics and Business Faculty of Economics and Finance: Uniwersytet Ekonomiczny we Wroclawiu Wydzial Ekonomii i Finansow, POLAND

## Abstract

Developing food supply chains in the African agriculture could be one of the keys for higher value-added activities and for the fair income of the stakeholders along the chains. Our research aims to investigate how these agricultural value chains are working in Northern Ghana and how to develop them. To estimate meat demand in the Tamale Metropolis, we carried out a large-scale survey with more than 300 interviews. Furthermore, we also measured the awareness of processed meat products. Based on the results, our conclusions are as follows: Development of public services offers the opportunity to (1) gaining market power for ourselves while losing market power for others, (2) indirect takeover of control on political and civil societies while losing control for others, (3) to win allies and friends on one hand, potentially losing allies and friends on the other. After spatial analyses of grazing areas, animal markets, trading routes and witnessing the descriptions of basic macroeconomic differences within Ghana; we must conclude that live animal trade is south-orientated, where traders are able to bargain higher prices. Due to northern locational advantages, the price of animals could be reduced. The presumably cheaper workforce and dozens of unemployed young males could also alleviate the financial burdens.

## 1. Introduction

The developing world has been targeted by foreign direct investments for many decades. These financial inputs are usually aimed at acquiring rights to exploitable natural resources (e.g., logging, mining) or in gaining the privilege to build and operate common utilities such as roads, bridges, or sewage treatment plants. Interest in farmland and agriculture has manifested over recent years due to the explosion of the world’s population and their demand for food and non-food agricultural products [1,2]. West Africa is the second most populated and rapidly urbanizing region of the continent after its Eastern segment [3]. To observe the last 30 years’ periodically collected data regarding the changing size of Ghana’s population, it is easy to notice that a constant increase has reached 28 million people at this time. Higher educational levels and better salaries act as breaks, correlated with a lower number of children [4].

In rural sites, agriculture as a labor intensive sector is the main platform of work. South Ghana mainly produces cash-crops such as cacao and cashew, unlike the Northern segment where main activities are maize-, millet-, yam-, groundnut plantation and animal rearing.

Following one of the World Bank’s publications on the topic ‘urbanization in Africa’ [5,6], we can declare that urbanization is driven by three clearly divinable factors: (1) rural push, (2) urban pull and the (3) urban push. These well-known differences between spatial developments are worth analyzing, not just for understanding the rural-urban relation, but in a regional context such as North and South Ghana or even among the whole of West Africa. Referring to numerous published articles, we can declare that Ghana is separated into two main parts economically, politically, infrastructurally, organizationally, and hierarchically [7–10]. Residual migration within West Africa could be the flow of labor, aimed at finding better paying or real and existing job opportunities. Escape from conflict-affected regions is also a sub-factor of movements [11–15]. During our research in Tamale, we met several families who have migrated to Northern Ghana from Northern Nigeria, either due to armed conflicts or prolonged unemployment. There are many young male pedicurists lining the streets, snapping metal tweezers at tribal rhythms, whose income from nail trimming at bus stops provides a good livelihood in Togo or Burkina Faso. The attractive environment of the city should make us think about the upcoming years’ development strategies where schools and job creation will need to be a highlighted part of planning; especially in slums and ‘zongo’ areas. Small urban and peri-urban family farms are also essential components of livelihoods. The sustainment of vegetable plantation, fruit harvesting and animal rearing within the city borders is vitally important for urban food supplies [16]. Demand in excess of domestic production may be covered by import, but to spend this high amount of money on foreign products, while the majority of rural youth is unemployed, seems an erroneously designated road to walk on. Developing rural areas through agricultural- and food production with related educational activities would be appreciable for everyone and could help to reach from Goal 1 (end poverty) to Goal 4 (equitable education) of the Sustainable Development Goals of Ghana by 2030 [17].

People usually prefer to cover their needs of a daily protein intake from livestock products. Ghana’s average wealthier economic level–compared to other West African countries–combined with an increasing population, result in an increasing demand for meat- and dairy products [18]. The food processing sector of Northern Ghana is dominated by small scale slaughterhouses and crop mills. Transportation of raw materials south is common practice as it is profitable in the short run. A good example of this is the presence of live animal transport in West Africa where animals grazed in Mali, Niger, Burkina Faso are driven once or twice a year to Northern Ghana and then transported by truck to the south [19]. As a case study of Kumasi Abattoir shows, cattle are transported from all over rural Ghana and some neighbouring countries also [20]. The fact that ruminants, especially cattle, are being grazed in the Sudano-Savannah zones, has to boost upcoming research and industrial movements to start focusing on the original breeding zones; what is the most effective way to cut off long distance transportation and establish local job opportunities.

Grazing in arid, less rainfed, areas and consuming food produce in wet tropical and sub-tropical areas raises concerns about the optimal management of our earth’s water resources. Development and application of the ‘Virtual Water Flow’ theory is named after the Dutch academician Arjen Hoekstra who developed a theory on the amount of commercially transported water required to produce the commodity [21]. This was applied to the food logistics of Tamale and Ougadougou–capital of Burkina Faso—by Akoto-Danso and colleagues who estimated the amount of live animals brought into Tamale per day to be 20 tons [22].

Karg et al., between 2013 and 2015, carried out wide-ranging research into Tamale’s and Ougadougu’s (capital of Burkina Faso) foodsheds and agri-products supply. A well-composed thematic research collection and the gorgeous collaboration of German and Ghanaian researchers afforded lecturers and universities the opportunity to produce a beautiful analyzation of eating habits and provide the first prediction of weighted city-imported foodstuffs/capita/day. Sources of livestock, frozen fish, vegetables, fruits and crops are visualized in an article published in 2016 [23]. Another paper of theirs points out the role of small-town markets; this specific measurement of rural-urban connections and level of development are well described [24] (. With movement of animals, not just raw material for the meat industry is transported, but also animal diseases across countries, regions and districts. As several Ghanaian authors have published about the topic over the last several years, there are detectable and economically palpable diseases spread among animals and humans [25–27]. Sub-species of *Escherichia coli* are the major bacterial groups which need prodigious attention [28,29], but *Salmonella and Staphylococcus sp*. are also occurring as emergent, harmful, microbe groups in the meat supply chain of the Tamale Metropolis [30–32]).

Our research aims to investigate how these agricultural value chains are working in Northern Ghana and how to develop them. In the first part of the paper, we provide an overview of the conditions for agricultural production in Northern Ghana, to help the better understanding of the difficulties of the sector we investigated. Within this context, we had the goals to draw the attention to the agricultural potential of the Savannah belt, in particular the meat sector and related educational activities and to collect a useful dataset, what could serve as fertile soil and starting point of further studies.

After introducing the methodology toolset we used, the results of the research are highlighted, followed by the conclusions, including the implications of the research results as well.

### 1.1. Conditions of Tamale, Northern Ghana

Ghana can be divided into two very different areas: Southern Ghana and Northern Ghana. Their differences lie not only in the periodically changing weather (in the south, there are two rainy and two dry seasons while in the north, there is a short rainy and a longer dry season), but also in their tribal-, religious diversity, and related traditions. As we are move away from the Golden Coast of the Gulf of Guinea and heading towards the North, conditions require a resilient person. Low humidity and unbearable heat are the results of infrequent rains. A well-known publication–“Traditional recipes from the Northern Region of Ghana” [33]—includes almost all the cultivated crops, vegetables, fruits, spices and domesticated animals that people traditionally prepare to eat and drink throughout their entire lives.

As we have mentioned, the fast-growing urban population is becoming less and less committed to any form of food production activity [4], but 45% of Ghana’s rural population is still engaged in agriculture [34] (. In the Economic Capital of Dagbon Kingdom, Tamale, only 20% of the population undertake any kind of agriculture-related work. Despite the fact that our city of study is surrounded by hundreds of communities, Tamale still maintains importance in transportation, trade, logistics and industry [35] Interesting fact, that since independence in March 1957, the traditional kingdom of Tamale (as much as all other such formations of Ghana) assumed customary role.

Tamale had been selected because of its

1. Geographical position [near the main West African pastoral zone; located at the main north-south national highway (N2) of Ghana]
2. Benefits of common utilities (constant electricity and water supply)
3. Easy accessibility via road and air (transportation)
4. Everyday operating markets (without break)
5. Knowledge material and educational centre (Meat Unit, University for Development Studies).

## 2. Research methodology

From April 2019 –December 2019, primary data collections were carried out mainly within the Tamale Metropolis and Nyankpala community. Due to the high Muslim population percentage, we had to avoid any food-related data collection in May and June because of Ramadan; denoting that Muslims are accordingly reducing their diet to include only tea and bread and consumption of any other food products (such as meat) diminish.

During the overall collection of data, we focused on the meat supply and value chain of the Tamale Metropolis.

To understand the basics of animal production and live animal flows, especially importation procedures and primary trading methods of livestock, we visited four governmental institutes to conduct casual interviews. Those institutes were:

1. Livestock Production Unit, Ministry of Food and Agriculture, Tamale Metropolitan Directorate
2. Veterinary Service, Ministry of Food and Agriculture, Bolgatanga Municipal Directorate
3. Border Entrance Point, Veterinary Service, Paga
4. Faculty of Agriculture, University for Development Studies, Nyankpala

Collected information led me to animal markets. Main trade activities, bulk purchases of ruminants are concentrated on these territories. Sites attended:

1. Savelugu Animal Market (middle size)
2. Tamale Animal Market (big size)
3. Buipe Animal Market (big size)

Observing the cattle transportation process, livestock was either transported one by one in small vehicles or in crowded trucks at speed throughout the country with fast delivery to slaughterhouses and meat manufacturers ensuing. Within the Tamale Metropolis and Nyankpala, the four main processing factories were: 1. Tamale Main Abattoir; 2. Gee’s Fresh Point; 3. Farm Gate; 4. Meat Unit, UDS.

Eleven cold stores in the Tamale Metropolis were also visited and employees interviewed by us.

To estimate meat demand in the Tamale Metropolis, we carried out a large-scale survey within the population. Demand of particular meat products was estimated (consumer side). Furthermore, we also measured the awareness of processed meat products.

In total, within one week (between the 30th of September and the 5^th^ of November), we interviewed 334 people in the centre of Tamale, around and inside the Main Market.

### 2.1. Data collection

#### 2.1.1. Primary data

We had the opportunity to join the UDS Meat Unit team for 7 months long. Here, we could see the basics of meat production technologies and trade. They brought me to Savelugu to buy animals and explain the complete methods of livestock trading, transportation and market periodicity. The level of meat production technologies, capacity of facilities, and basic data about production yields were measured and collected. We discussed technological utilization and use of local materials like spices, fats and oils. As far as our situation allowed it, we tried to assess the limitations of production facilities, utility gaps, and locational advantages and disadvantages. First, we designed a quantitative, semi-structured, questionnaire and successfully interviewed the 4 biggest slaughterhouses/manufacturers (Tamale Abattoir; UDS Meat Unit; Gee’s Fresh Point; FarmGate) to map out local industrial scale. First of all, we asked them to answer 9 questions about daily operations, type of activities (slaughterhouse/boning plant/further processing facility), production quantities, animal supply and origin. The number of employees and their levels of education were also recorded. Every time we visited a firm, the focus was set on specific topics and strictly noted the answers. These topics were:

1. Origin of animals
2. Processing technologies, processing level of products, storage methods
3. Distribution and main markets
4. Employees, education level
5. Advantages and disadvantages of Tamale as an industrial centre

Secondly, one semi-structured, qualitative questionnaire for 11 randomly selected cold stores (including 15 questions) was designed to measure the origin of products, the quality and reliability of local manufacturers, biggest consumers and the distribution system. During our dialogues, it was possible to shine a light on some specific trade habits, consumer preferences and stores/refrigerator capacities. To complete our insight into the last part of the supply chain, we reached out to 334 randomly selected inhabitants around Tamale Main Market with a finely structured questionnaire (including 21 questions) to measure special weekly meat consumption and knowledge about origin and preference of meat products. First of all, we asked them to indicate their weekly number of breakfasts, lunch, supper and snack intakes. They had to indicate whether beef, chicken, fish, and pork. Secondly, we were interested in their knowledge about the origin of meat products (domestic or imported), and their shopping habits within the city. The final few questions were designed to garner an idea about the storage of purchased meat. All questions were meat related.

#### 2.1.2. Sources of secondary data

Portions of secondary data were collected from a Tamale-based association named ‘Ghana Developing Communities Association’ to indicate the scale and role of animal markets and, furthermore, the ‘South orientated’ live animal trade. Meat Unit, the meat manufacturer of the University for Development Studies in Nyankpala, provided data on cattle carcasses and offal weights. Estimation of population and other socio-related data was provided by the United Nation Data Base and FAOStat.

## 3. Results

To prove the credibility of this publication, we compared data published in Population and Housing Census 2010 by the Ghanaian Government to the descriptive consumer information collected by us (Table 1).

**Table 1:** Statistical comparison of population and Housing Census 2010 and the current research. (Edited and collected by the authors).

		Census in 2010	Survey in 2019
Age (under 30 years)		63,9	65,0
Major tribe (Dagomba)		52,7	56.9
Major religion (Islamic)		60,0	71,9
Average household size		7,1	7,5
School attainment	Non-formal	56,6	30,2
	Primary	22,9	4,2
	High school	17,8	46,1
	Tertiary	2,7	19,5

The only conspicuous difference appearing is with school attainments. It seems that educational levels have been growing. This may be resultant of the implementation of a free high school system by the government in 2017.

### 3.1. Role of animal markets

Small town markets are the first window of opportunity where farmers introduce their food products to wholesale or retail businesses. Big ruminants like cattle are usually trekking the whole year around West Africa (Niger, Mali, Burkina Faso) and twice a year reach North Ghana in what seems as a highly appreciated market because of its constantly solvent demand and relatively well-operated meat industry.

#### 3.1.1. Spatial location, monthly livestock flow and time series analyzation of pointed animal markets

**Fig 1:**
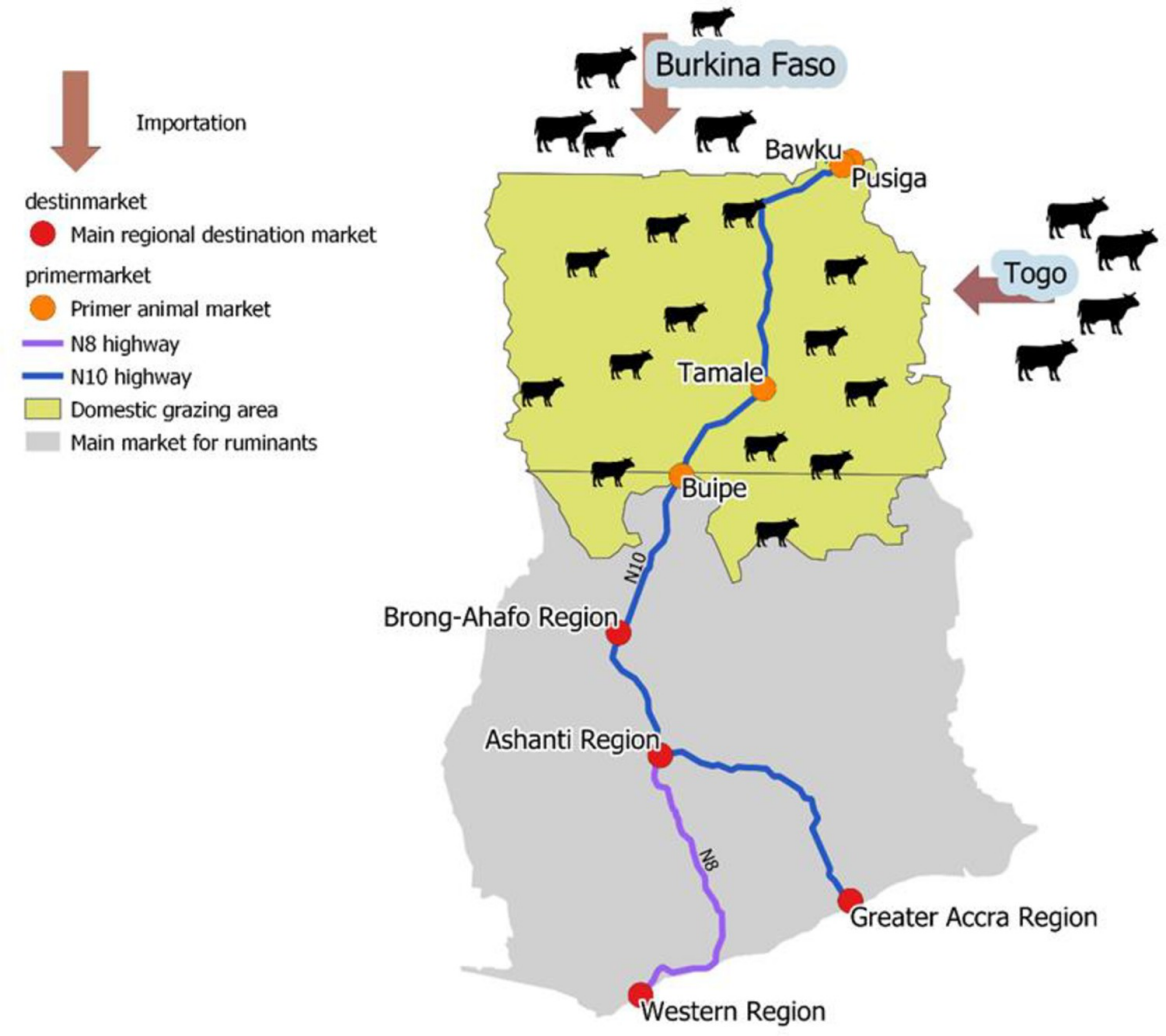
The South orientated livestock transportation through Ghana (map edited by the authors).

Four primer wholesale northern animal markets were mapped out to indicate southern-orientated live animal transport. The importation of livestock is usually originating from Burkina Faso and Togo. The green area indicates major domestic production of ruminants (North Ghana), while the grey area shows usual destinations and solvent demand (south Ghana). The N10 main road deserves special attention in terms of food transportation (Fig 1).

**Fig 2:**
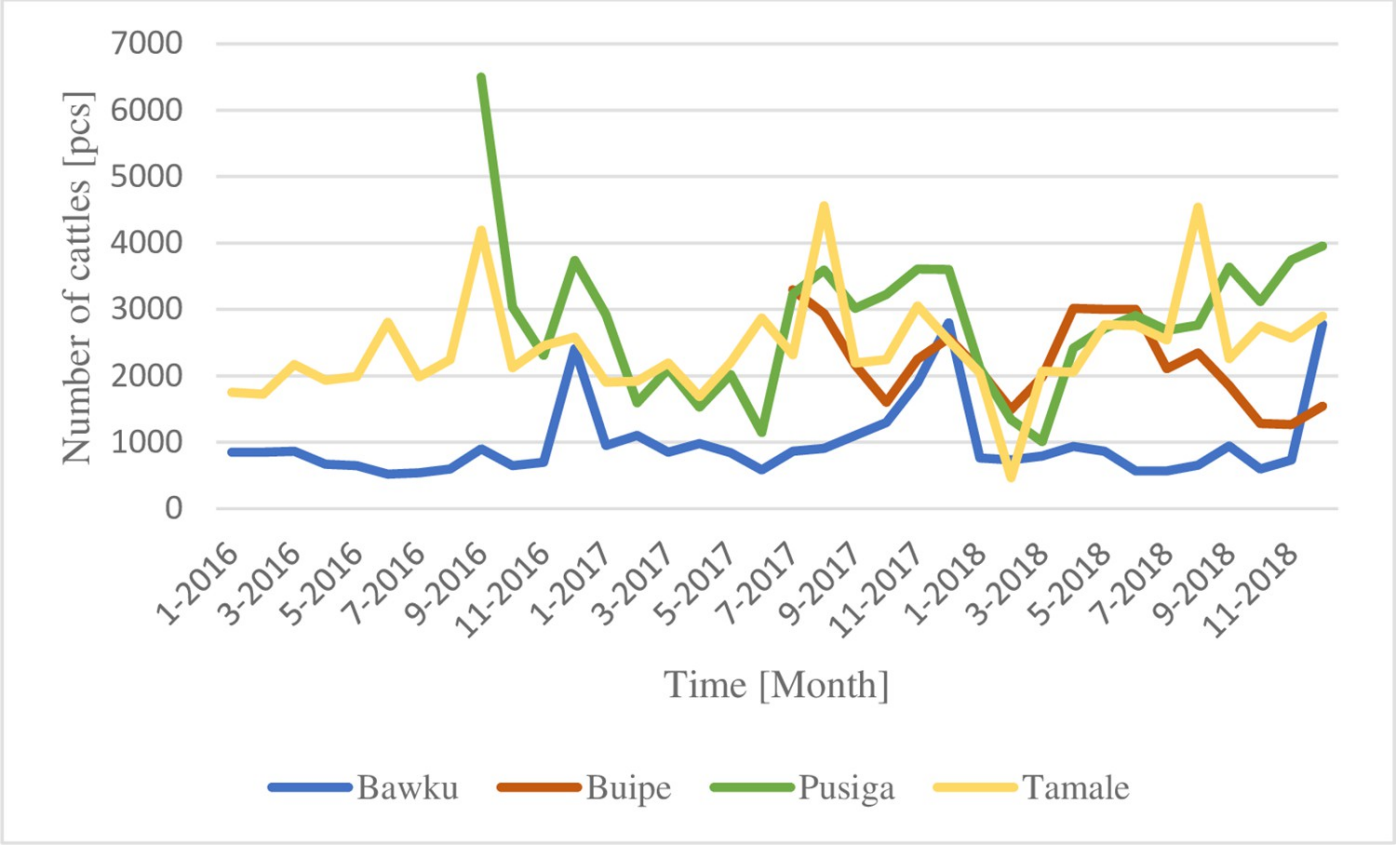
Monthly presented number of cattles in four North Ghanaian animal markets (own edition).

**Fig 3:**
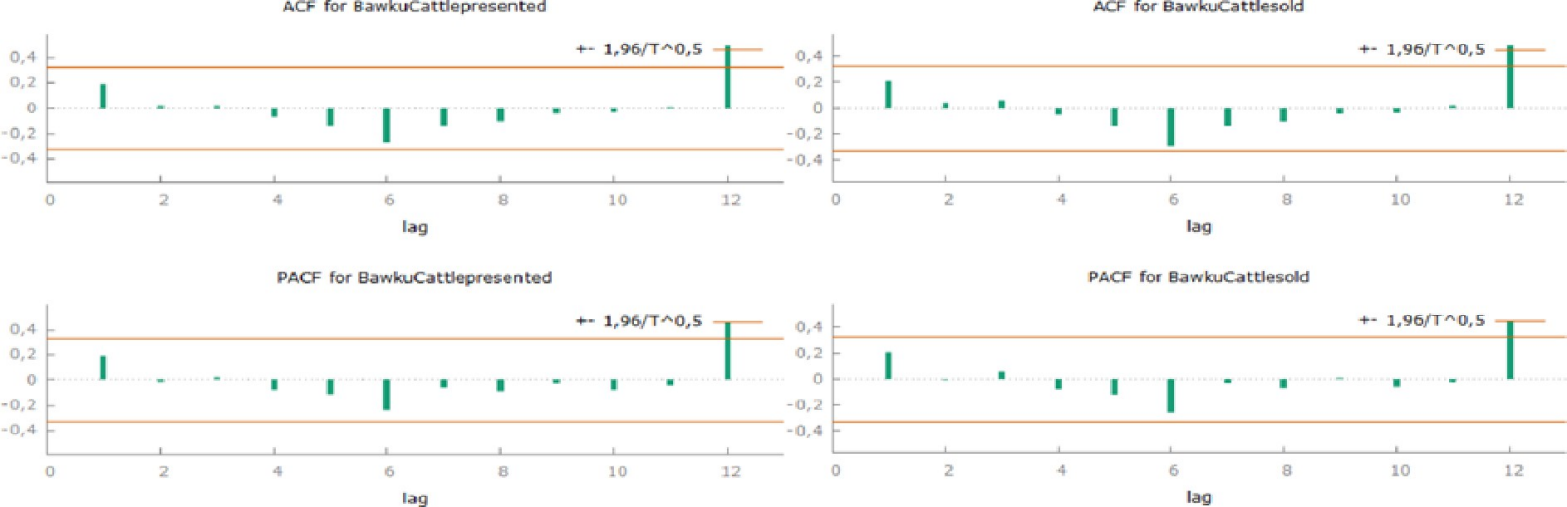
Correlogram of monthly presented and sold cattle in Bawku (own edition).

Consequently, the reasons why we did not want to attach the markets together and present it as one whole dataset: 1) There are huge differences between markets in the number of presented cattle. It is worth seeing the gaps between them; 2) Periodical shifts between markets are noticeable in Figs 2 and 3) Before we will have enough data (at least 30 months/market), it is not acceptable to run them through any statistical analyzation. Only the datasets from Tamale and Bawku hit the bar with its 36 impulses.; 4) Four animal markets do not cover the entire north Ghanaian cattle trade existence.

Huge differences between size of markets can be determined around Tamale. As the main northern city with a market operating every day, gives rise to a relatively high turnover of cattle during the whole year. Pusiga as the first trade point in the north-western tip of Ghana, also varies from 1,000 up to 6,500 heads of cattle.

Every Twelfth month peak, in December, represents Christmas. Bawku shows the most outstanding values currently, although Christianity is not completely obvious there. A correlogram for Bawku’s dataset shows a significant (p<0,01) autocorrelation within every twelve months. A partial autocorrelation outlines almost the same values, so we can declare that both datasets about Bawku’s cattle market (presented and sold) are recurring (Fig 3).

**Fig 4:**
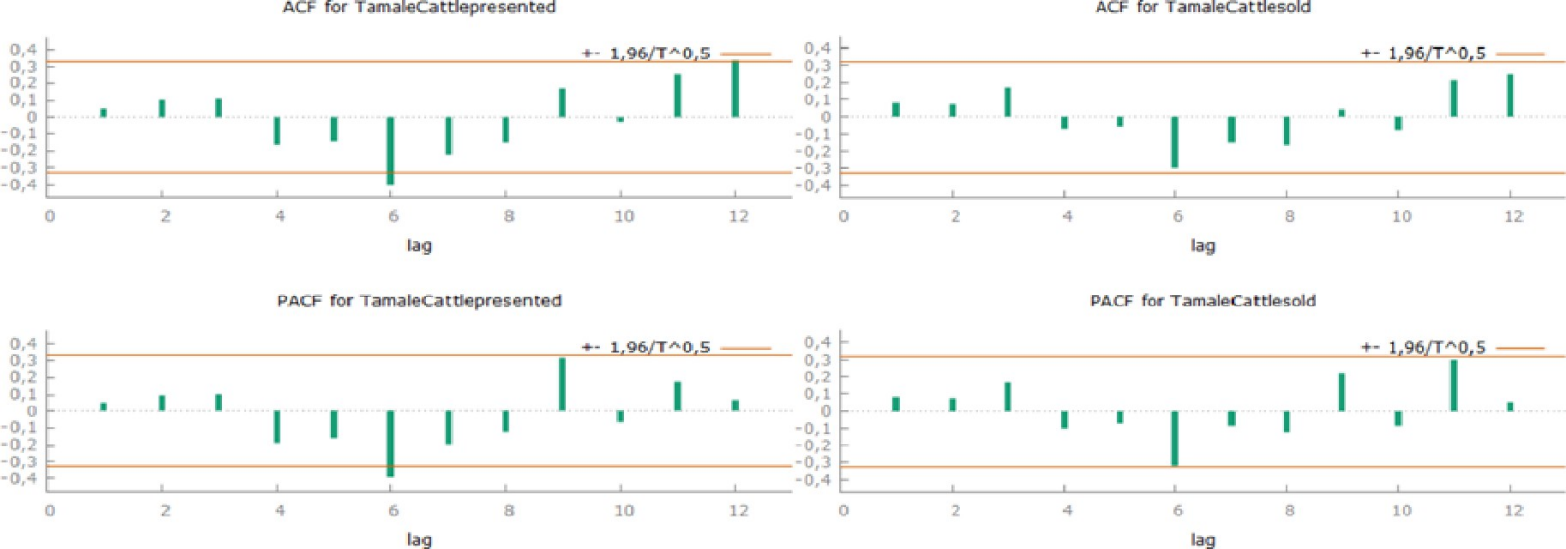
Correlogram for monthly presented and sold cattle in Tamale (own edition).

The Tamale cattle market correlogram denotes that every 6th and every 12th previous data correlate with each other. Both autocorrelation and partial autocorrelation in slip level 6 show a negative autocorrelation. That means, data with 6 months’ difference between them are inversely proportional to each other. It could refer to the changing of seasons and also religious holidays. Yearly periodicity of the market would also mean a predictable variance, but partial autocorrelation does not verify it (Fig 4)

**Fig 5:**
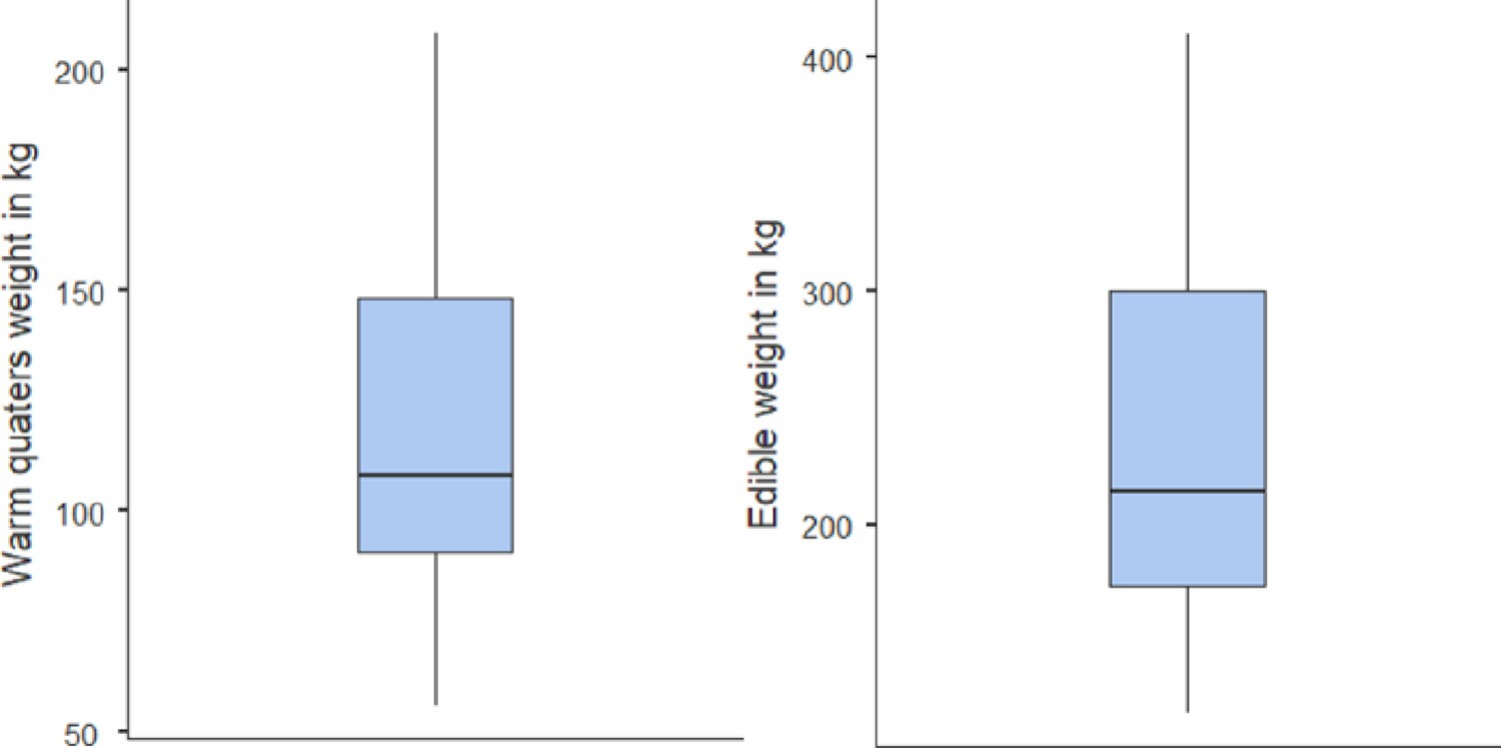
Boxplot diagrams of technologically useful weights (own calculation).

Average difference between the means is 116 kg. This equals the summary weight of head, legs and internal organs. Both datasets are distorted. Skewness and kurtosis show abnormal distribution as per the Shapiro-Wilk-test with p-value lower than 0,01. In the case of random choice, positive skewness means that our chance is higher in finding a carcass below the mean weight (representing 120 kg and 236 kg). Median is the ‘middle’ value in the list of carcass weights which shows us where the middle value is in the wide range of data. If it occurs below the mean, it means that carcasses are usually lighter rather than heavy weighted (medians are 108 kg and 215 kg). Kurtosis measures the tail heaviness of the distribution, which shows negativity in both cases (-0,538 and -0,927), what means the distribution curve is wide and heavyset. Cattle carcasses and live weights vary in wide range due to different levels of their maturity and types of breeds such as Zebu, West African Short Horn and Sanga (Teye GA and WK Sunkwa, 2010). (Fig 5)

Our main goal was to give a description, the relationship between ‘live weight’ and ‘technologically useful weight’. Hide blood content and weight had been also added to ‘edible weight’ under the term ‘live weight’. According to the Ministry of Agriculture and Rural Development, South Africa, hide weight in terms of cattle in percentages of live weight is about 7% (Ministry of Agriculture and Rural Development, Province Kwazulu-Natal, South Africa). Blood content of matured cattle in percentages of live weight varies. An article also from South Africa (Agbeniga and Webb, 2012) indicates average blood loss to be 2.4% of the live weight, while another from Turkey shows 3.1% in the case of conventional stunning and eviscerating (MH Anil et al. 2006). We have taken the average of the two and calculated with *2*.*75% blood content*. *Equalization between warm quarters weight and live weight is as follows***:**

y=1,9995x+20,154

$$R^{2}=0,9431$$

With this model, we can plan, estimate and calculate the needed number of cattle to satisfy the beef demand of Tamale’s population. Pictures from the Meat Unit are attached in Appendix 2.

### 3.2. Quantities of meat production and consumption in the Tamale Metropolis

#### 3.2.1. Estimated consumption quantities /personal survey/

To extract a weekly portion of consumed meat by type, we have summarized the answers provided by every single consumer. Main meat types of our interest were beef, chicken, fish and pork. Calculation of exact portion from the given intervals (Table 2):

**Table 2:** Aggregation weekly meat intake.

	Times per week				
**Breakfast**	0	1x	2-4x	>4x	NA
**Lunch**	0	1x	2-4x	>4x	NA
**Supper**	0	1x	2-4x	>4x	NA
**Snack**	0	1x	2-4x	>4x	NA

0 means 0; 1 means 1; 2–4 means 3; >4 means 6.

**Fig 6:**
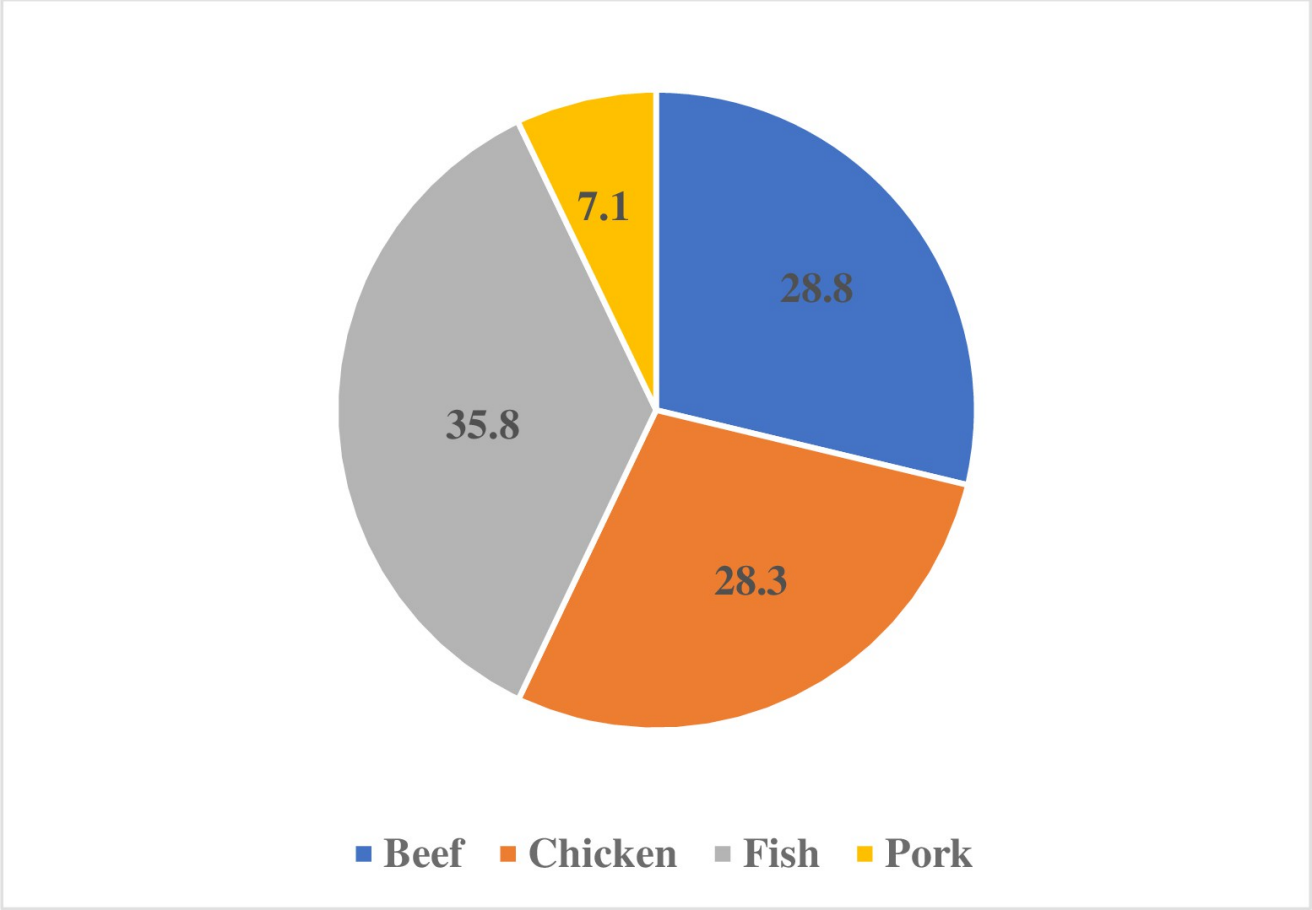
Distribution among consumed meat in daily diet in percentages (own edition).

The fact that 334 persons had filled out the survey, enabled us to divide the cumulative numbers to see the average portion of consumed meat by type and meal. Snacks do not play a major role in the diet of Tamale men; frequencies of answers were too low to calculate with. (Fig 6)

Consumed portions of different meat types were also calculated (Table 3). Now, we should figure out the average grams of portions. we took three pieces of cooked/fried meat from every type and weighed them. One piece of meat is shown as being among the following average weights: beef– 25g; chicken-50g; fish-25g; pork-50g. During our time in Ghana, every time we ate in Ghanaian society, we consistently counted the pieces of meat on the plate. Beef is shown as 2 pieces, chicken shown as 1, fish shown as 2 and pork shown as 1. Multiplying the portions with the pieces, we came to an averagely-consumed number of cooked grams of meat per capita. To be able to calculate the raw meat demand per person, we had to figure out the percentage of cooking loss. We were also involved in some kitchen work, and cooking losses showed variation between them. Chicken and fish have lost averagely 15% of their raw weight during deep frying. Pieces of beef and pork were tougher, and losses were no more than 5%. With this calculation method, raw meat demand seems like an available data for further studies.

**Table 3:** Calculation of raw meat demand by types (self calculation).

Estimated raw meat demand by type in gramms					
	portion/capita/day	gramm/portion	gramm/day	cooking loss (%)	rawmeat gramm/capita/day
**Beef**	0,85	50	43	5	45
**Chicken**	0,84	50	42	15	48
**Fish**	1,06	50	53	15	61
**Pork**	0,21	50	11	5	11

**Fig 7:**
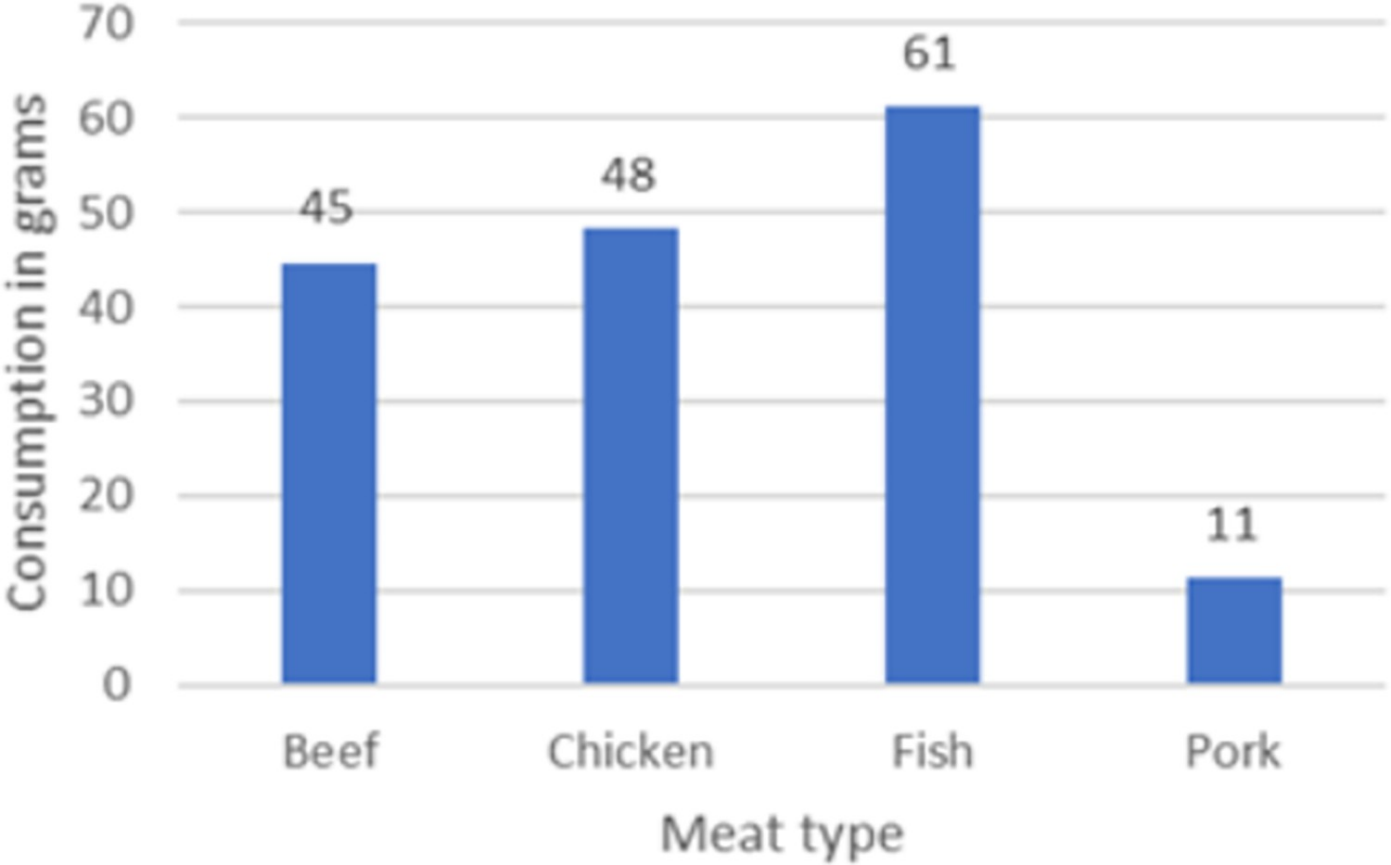
Raw meat consumption in grams per day per capita by meat types (self calculation).

Raw fish demand reaches the highest level among the four meat types we have measured. It shows 61 grams per capita per day. Beef- and chicken-meat share second place—closely equal at 45 and 48 grams. Low-level pork consumption (11 grams/capita/day) indicates that Tamale is mainly populated by Muslims (Fig 7).

To extrapolate these differentiated portions’ quantities for the whole population of Tamale (523.568), we need to multiply portion’s weights with the size of population. Because we are assuming that consumer habits did not change between the survey time (2019) and nowadays (2020), daily quantities of raw meat demand are estimated as indicated on Table 4:

**Table 4:** Daily accumulated raw meat demand in Tamale (self calculation).

Tons of raw meat consumed by Tamale population/day	
**Beef**	23,4
**Chicken**	25,2
**Fish**	32,0
**Pork**	5,9
**Total**	86,5

#### 3.2.2. Estimated production quantities /factory visit/

In this section, we are going to represent production quantities, focusing on beef production. Beef is our main interest due to

- it’s acceptation by every religious group
- forms of a relatively large size which could serve as material for further processing
- cattle are adapted to the savannah climate

Production quantities are varying between manufacturers. For example, Tamale Main Abattoir deals only with cattle and small ruminant slaughtering, while Farm Gate and UDS Meat Unit provide a wide range of processed meat products. Table 5 shows the weekly number of slaughtered animals by type per firm.

**Table 5:** Number of slaughtered animals/week in Tamale (own collection).

	Cattle	Chicken/Guinea fowl	Sheep/Goat	Pig
**Main abbatoir**	350	0	100	0
**UDS Meat Unit**	3	30	2	2
**Farm Gate**	0,5	150	0,5	0
**Gee’s Fresh Point**	0	400	7	0

**Fig 8:**
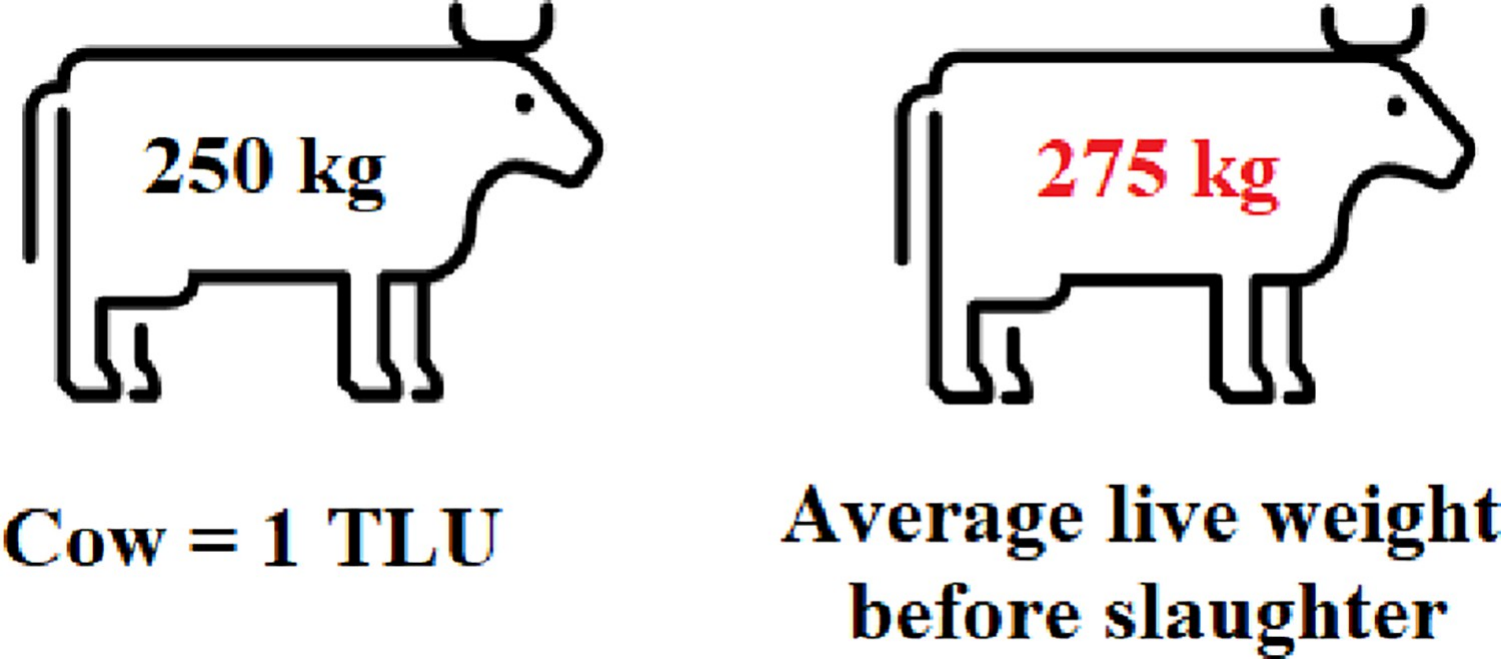
Average weight of cattle before slaughter in Tamale (self calculation).

As a case study from Kenya published in 1991 by the Food and Agricultural Organization (Kassam et al., 1991) shows, domesticated tropical animals can be determined using the Tropical Livestock Unit. Herd structures have been defined in terms of number of heads of animals as well as in terms of referencing what Tropical Livestock Unit (TLU) defined as a mature animal weighing 250 kg. Surprisingly, our own calculated dataset, collected from the University for Development Studies, shows a slightly higher live weight of cattle (Fig 8).

The aggregated beef (edible weight) production quantity of Tamale is estimated to be 89 tons/week (Table 6). Numbers depend on the 4 main slaughterhouses’ declarations.

**Table 6:** Scale of weekly cattle slaughtering within Tamale in kilograms (own calculation).

	Number	Live weight (kg)	Edible weight (kg)	Warm quarters weight (kg)
**Main abbatoir**	350	0	100	0
**UDS Meat Unit**	3	30	2	2
**Farm Gate**	0,5	150	0,5	0
**Gee’s Fresh Point**	0	400	7	0

#### 3.2.3. Deficit of production

Comparison of our processed quantitative data between beef production and consumption outlines a relatively high production deficit. The following tables (Tables 7 and 8) show the daily deficit of beef in tons and lacking number of cattle.

**Table 7:** Daily deficit of beef production in tons (own calculation).

Produced	Consumed	Deficit
12,7	23,4	***10*,*7***

**Table 8:** Monthly lack of cattle supply (self calculation).

Beef production deficit in kgs	Average cattle weight in kgs	Lacking number of cattle
321 000	275	***1 168***

## 4. Conclusions and limitations

In today’s sense, when local events become global within a second, the construction of a secure and robust social system cannot ignore public service. Not just bad recipes or harmful religious and political ideologies are sweeping through the world, showing extremities in the light of goodness, but both conventionally and revolutionary solutions for local problems are popping up. What could we share with the world if we have nothing to show? Development of public services offers the opportunity to (1) gaining market power for ourselves while losing market power for others, (2) indirect takeover of control on political and civil societies while losing control for others, (3) to win allies and friends on one hand, potentially losing allies and friends on the other. The innumerable frontlines illustrate a labyrinth that could lead us to create and follow a bad strategy with ease, where dead ends appear in the forms of commodity duties, embargos, increased military presence, bioterrorism or even disrupted food security. With well-designed and structured decision making, we could have something to offer.

First, we would like to turn the focus on the credibility of this paper. Descriptive statistics of the accomplished consumer analysis allow us to see the 2019 surveyed population shows a similarity to the published data by Ghana’s Government in the 2010 Housing Census. Not just the estimation of current consumer eating habits and basic preferences, but later research and development strategies could be planted into this ground bedded by us.

Secondly, we need to add supplements to the sections which deal with the quantitative dataset of consumption. Unfortunately, chevon and mutton, as major sources of animal-based protein, were not measured in the consumer survey. It is a problem because people in Northern Ghana, especially Muslims, highly appreciate these types of meats. Addition of these data would result in a more accurate estimation of consumption. The currently measured 60.2 kg annual meat intake per person seems like an over-calculated estimation compared to the 9.32 kg published by FAO in 2017. Knowing Ghanaian food recipes which do often contain more than one type of meat (almost all stew types include smoked or dried fish e.g. fish bra leaves soup) should remind us to gently handle consumption data although we can declare that an effective traceability system does not exist in Ghana. For this reason, macroeconomic essays are not able to provide as accurate measurements as microeconomic research can do. Referring to Karg (2016) and Akoto-Danso (2019), the daily incoming quantity of livestock to Tamale in live weight per person is about 100 grams. That means, annually, 33.26 kg of edible weight. In addition, the production output of urban and peri-urban animal husbandry of Tamale and the trade quantity of frozen meat products by cold stores would result in the total meat supply of Tamale. In this way of thinking, *annual meat consumption within the Tamale Metropolis per person is estimated to hold a value between 33*.*3 kg and 60*.*2 kg*.

As Tamale is showing this high-volume consumption on its own, a large part of the supply arrives not just from outside of the city, but outside of the country; the time has come to start thinking about long-term collaborations between local and foreign knowledge.

In the case of red meat production, the location of Tamale compared to other major southern cities (e.g. Accra, Kumasi, Sekondi-Takoradi, Sunyani, Techiman etc.) seems definitely more advantageous. As we are farther and farther from the south, we are also getting closer and closer to northern pastoral and grazing areas of cattle and small ruminants. After spatial analyses of grazing areas, animal markets, trading routes and witnessing the descriptions of basic macroeconomic differences within Ghana; we must conclude that *live animal trade is* **s***outh-orientated*, *where traders are able to bargain higher prices*. *Due to northern locational advantages*, *the price of animals could be reduced*. *The presumably cheaper workforce and dozens of unemployed young males could also alleviate the financial burdens*. *Cattle have received a deeper overview because of their large meat mass and proven meat production technologies that could be implemented within local conditions*. All other calculated models between cattle’ live weight and technologically-useful or ‘edible weight is aimed to help further planning. With the published models, we can estimate the needed number of cattle for sufficient production.

Actually, neither agricultural- nor food-production can stand on one leg. A knowledge-equipped and practiced, skilled, workforce, technological and infrastructural development, make up large parts of a successfully-operating firm.

First, *technological development* should be aimed at avoiding diseases and contaminations spread through blood, faeces, and other internal fluids. The technological transformation of the floor (e.g., metal lattice instead of adjacent tiles) needs to be implemented to secure a long shelf life of products. Under other infrastructural and technological development, we mean construction of high-capacity cold stores because the thirty years old method of producing corned-canned beef by Zuarungu Meat Factory in nearby Bolgatanga–a product that does not need cooling—cannot compete with contemporary popular sausage products which do need a cool environment.

*Establishment of an Agricultural Vocational School*, where–among various professions–*butchering should be highlighted*. In Dagomba culture, butchers (Nakohama) form a highly appreciated group of people. In the Dagomba Kingdom, chiefs always keep a butcher within the community elders. Butchering, as an ancient profession, needs tiny reform to serve Ghana. Hygienic knowledge and practical skills like cutting and boning need to become substantial training elements. For adaption to changing technologies, *meat-technologists and mechanics are suggested to be trained within the Meat Unit*, *at the University for Development Studies*.

*Scholarship programs offered by the European Union (EU) have to be reformed into traineeships*. Dual tertiary education means students are engaged 50–50% by an educational institute and a company. Students should receive a monthly scholarship by the EU (any public institution), but the whole wage of the traineeship should be transferred in one instance at the end of the program (by the company). The student (after returning to Ghana) has to invest this amount of money into one of those agricultural practices that he/she has learned in the EU. The process need to be controlled by supervisors.

*Collection of different data about animal markets and stock flows*, *meat-manufacturers*, *cold stores*, *dressing percentages*, *structure of education* are all lined up in the queue of Tamale’s advantages. Upcoming development strategies have to include plans like:

1. Sustainment of grazing grasslands in the pastoral zones
2. Operation of modern, food demand led agricultural-vocational schools; reformation of tertiary scholarship programs
3. Transfer of well-proven meat-manufacturing technologies

## Supporting information

S1 File AnMKTtimeseries. (XLSX)

S2 File Bawku Livestock msrket Monitoring data. (XLSX)

S3 File Buipe Livestock msrket Monitoring data. (XLSX)

S4 File carcassweightMeatUnit. (XLSX)

S5 File Factories. (XLSX)

S6 File HibbaConsum. (XLSX)

S7 File Pusiga Livestock market monitoring data. (XLSX)

S8 File quest1factories_skdedits David finished (1). (DOCX)

S9 File questMeatCon. (DOCX)

S10 File Tamale Livestock market Monitoring data. (XLSX)
