# Correction: Opportunities within the meat supply chain in Africa—The case of beef production in Northern Ghana

**DOI:** 10.1371/journal.pone.0298803

**Published:** 2024-02-08

**Authors:** Áron Vaskó, Imre Vida, László Vasa, Frederick Adzitey

## Notice of Republication

This article was republished on February 1^st^, 2024, to correct the author list and add Frederick Adzitey as the fourth author. Please download this article again to view the correct version. The originally published, uncorrected article and the republished, corrected articles are provided here for reference.

## Supporting information

S1 File Originally published, uncorrected article. (PDF)

S2 File Republished, corrected article. (PDF)
