# Supplementary material for: Opportunities within the meat supply chain in Africa—The case of beef production in Northern Ghana
Source: PLoS One. 2022 Jan 18;17(1):e0260668. doi: 10.1371/journal.pone.0260668 (PMC8765630; doi:10.1371/journal.pone.0260668)
Supplement: S8 File — (DOCX) [file pone.0260668.s008.docx]

Questionniare #1

FACTORIES, TRADERS, DISTRIBUTORS

This questionniare is in colloboration with the Szent Istvan University, Budapest; University for Development Studies, Tamale and the Embassy of Hungary, Ghana. I am **Aron David Vasko**, MSc student in the field of **Food-processing and Economics**. This questionnaire is designed to collect information about **Animal Production** in the **Northern Region of Ghana** with a sharper focus on the **meat supply-chain**. The following questions will explore your company’s **products, variety and their production numbers**. It will also will also explore the number of employees hired and their working hours to get a clearer picture of the current **employment situation in the meat industry in Tamale**. The Embassy of Hungary in Ghana is interested in this work and will like to offer some support and/or investment in the indusrty based on the information provided by you. Accurate information is required from you to give a true reflection of the actual situation for the right support when necessary.

I appreciate your time and assistance in advance.

Sincerely yours,

Aron David Vasko

+233 55 37 35 731

1. **Please circle any area or areas which your company\shop deal(s) with:**
   1. Slaughtering
   2. Transportation of carcasses
   3. Processing of meat into products
   4. Trading (with final consumers)
   5. Distribution
2. **Please circle all the species that your shop\company supply\sell:**
   1. Cattle
   2. Poultry (guinea fowl, chicken etc.)
   3. Pigs
   4. Goats
   5. Sheep
   6. Others, please specify.........

# 1st Part, SLAUGHTERING

1. **How far do you carry animals to the slaughterhouse? You can circle more if it is necessary.**
   1. The company owns livestock just next to the factory
   2. 0-10 km
   3. 11-50 km
   4. 50-100 km
   5. 100-400 km
   6. 400+ km
2. **Where do you buy live animals? Please indicate your answer with an ’’X’’**

|  | Cattle | Pig | Poultry | Goat | Sheep | ____ |  | ____ |
| --- | --- | --- | --- | --- | --- | --- | --- | --- |
| Individual farms |  |  |  |  |  |  |  |  |
| Tamale Animal market |  |  |  |  |  |  |  |  |
| Bowku Animal market |  |  |  |  |  |  |  |  |
| Savelugu Animal market |  |  |  |  |  |  |  |  |
| Buipe Animal market |  |  |  |  |  |  |  |  |
| Gushegu Animal market |  |  |  |  |  |  |  |  |
| Katinha Animal market |  |  |  |  |  |  |  |  |
| _________________ |  |  |  |  |  |  |  |  |
| _________________ |  |  |  |  |  |  |  |  |

1. **Where have theese animals produced? Please list the countries below.**
   1. Ghana
   2. Other:______________________________________________________________
2. **How many animals do you slaughter per week?**
   1. Cattle:.......
   2. Poultry (guinea fowl, chicken etc.):.......
   3. Pigs:.......
   4. Goats:.......
   5. Sheep:.......
   6. Others, please specify.........
3. **Who is responsable for the live animal transportaion?**
   1. The farmer
   2. The slaughterhouse
   3. Both
   4. Others, please specify.........
4. **Do you deal with ’’contract slaughtering”?** ( Your partner brings the live animal for slaughtering and you sell the carcass back to him\her.)
   1. Yes, all of the producement is contract slaughtering.
   2. Yes, majority of the producement is contract slaughtering.
   3. Yes, we deal with it sometimes.
   4. No, we do not do contract slaughter.
5. **Who brings animals for contract slaughter?** Plesase circle them below!
   1. Private butchers
   2. Other factories
   3. Households
   4. Individual farmers, who do not trade
   5. Other, please specify: ..........

# 2nd Part, PROCESSING

1. **Which type of unprocessed meat (bone-in, boneless) do you produce? Circle all that applies.**
   1. Beef
   2. Poultry
   3. Pork
   4. Chevon
   5. Motton/Lamb
   6. Others, please specify.............
2. **Which** **type of finished product(s) do you produce?**
   1. Beef
   2. Poultry
   3. Pork
   4. Chevon
   5. Mutton/Lamb
   6. Others, please specify...............
3. **What type of finished-meat products (cooked-smoked sausages, dried sausages, hamburger meat, etc.) do you produce?**  Please identify (A: Beef sausages, B: Beef garlic sausages, C: Beef Frankfurties, D: Beef hamburger meat, E: Beef pepperoni, F: Poultry sausages, G: Poultry Frankfurties, H: Poultry bacon, I: Pork Bacon etc.) and put them in order! If your variety is different than listed above feel free to add/change it!
   1. (*Product with most sales*):___________________________________
   2. *( Product with second highest sales):___________________________*
   3. *( Product with third highest sales)*:_____________________________
   4. *(Product with fourth highest sales):* ___________________________
   5. *(Product with fifth highest sales):* _____________________________
   6. *( Any other important products):* _____________________________
4. **Please indicate the average number of kilograms of sold products** **per day in the table below.**

|  | **Whole carcass** | **Unprocessed meat** | **Finished product** |
| --- | --- | --- | --- |
| **Beef** |  |  |  |
| **Poultry** |  |  |  |
| **Pork** |  |  |  |
| **Mutton** |  |  |  |
| **Chevon** |  |  |  |
| ________ |  |  |  |
| ________ |  |  |  |
| ________ |  |  |  |
| ________ |  |  |  |
| ________ |  |  |  |

# 3 rd part, DISTRIBUTION

1. **Who are your biggest customers?** (People who you directly sell to.) **Please list them if it is possible!**
   1. Other manufacturers (private butchers, other producers): _____________________________________________________________________
   2. Households: _____________________________________________________________________
   3. Traders (cold stores, shops, ect.): _____________________________________________________________________
   4. Distributors (who are not employeed by the company): _____________________________________________________________________
   5. Catering services, restaurants: _____________________________________________________________________
   6. Others, please specify...........................................................................................: _____________________________________________________________________
2. **Do you take pre-orders?**
   1. Yes, all of our production is based on pre-orders; we do not produce for storage. (Consumers are not able to buy randomly).
   2. Yes, majority of our production is based on pre-orders but we produce for direct selling too. (Consumers are be able to buy as walk in customers at random, but just from a narrow variety of products).
   3. Yes, small number of our production is based on pre-orders. We usually produce for random consumption. (Consumers are able to walk into our shop and buy randomly from a wide variety of products).
   4. No. All of our production is based on random consumption and walk in customers.
3. **How many kilogramms of production is based on pre-orders per day?**

|  | **Whole carcass** | **Unprocessed meat** | **Finished product** |
| --- | --- | --- | --- |
| **Beef** |  |  |  |
| **Poultry** |  |  |  |
| **Pork** |  |  |  |
| **Mutton** |  |  |  |
| **Chevon** |  |  |  |
| ________ |  |  |  |
| ________ |  |  |  |
| ________ |  |  |  |
| ________ |  |  |  |
| ________ |  |  |  |

1. **How often do your consumers make pre-orders?** Plesase circle the letter! (A.Always; B.Often; C.Moderate; D.Rarely; E.Never)
   1. Households: A B C D E
   2. Traders ( shops, cooled stores): A B C D E
   3. Other factories ( private butchers, other producers): A B C D E
   4. Restaurants, catering: A B C D E
   5. Other types of customers not listed above: ___________ A B C D E
2. **Do you offer distribution?**
   1. Yes
   2. No
3. **If yes, how do you do your distribution?**
   1. The company employs a distributor in full-time job.
   2. The company employs a distributor on a part-time basis.
   3. The company has a contract with an external distributor.
4. **How often and who do you deliver to? Please indicate your answer with an ”X”**

|  | Daily | 2-3 times\week | Weekly | Infrequent | Never |
| --- | --- | --- | --- | --- | --- |
| Households |  |  |  |  |  |
| Restaurants |  |  |  |  |  |
| Other factories, industries, butchers |  |  |  |  |  |
| Shops, cold stores |  |  |  |  |  |
| Others:…………………… |  |  |  |  |  |

1. **How do you deliver?**
   1. Meat van
   2. Motorbike
   3. Motor-king
   4. Taxi or public transport
   5. Others, please specify:.......
2. **Do you deliver the products in cooled environment?**
   1. Yes
   2. No
3. **Do you sell to somewhere outside of Tamale?**
   1. Yes, please specify: _____________________________________________________________________
   2. No

# 4th Part, GENERAL QUESTIONS

1. **How many people do you employ on a full-time basis? (At least 4 days a week and 6 hours per day)**
   1. 0-5 employees
   2. 6-10 employees
   3. 11-15 employees
   4. 20+ employees
2. **How many people do you employ on a part-time basis? ( Maximum 3 days a week and 6 hours per day)**
   1. 0-5 employees
   2. 6-10 employees
   3. 11-15 employees
   4. 20+ employees
3. **How many people are formal-educated in food-industry within the company? ( butchers, technicans, degree in any field of agriculture etc.) Please provide the qualification as well.**
   1. 0-5
   2. 6-10
   3. 11-15
   4. 20+
4. **Do you know of any secondary agricultural schools in Tamale?** **If yes, please list the names below!**
   1. ______________________________________________________________
   2. ______________________________________________________________
   3. ______________________________________________________________
   4. ______________________________________________________________
5. **Do you make documentaion on bought and sold animals, carcasses and meat products?**
   1. Yes, it is public for you
   2. No
6. **Please add your contacts below (Optional)!**

Company’s name:___________________________________________________________

Adress:___________________________________________________________________

E-mail adress:______________________________________________________________

Contact person:_____________________________________________________________

Phone number:______________________________________________________________

1. **If you have any comments or remarks, please note it below!**

**_____________________________________________________________________________________________________________________________________________________________________________________________________________________________________________________________________________________________________________________________________________________________________________________________________________________________________________________________________________________________________**
