# Supplementary material for: Opportunities within the meat supply chain in Africa—The case of beef production in Northern Ghana
Source: PLoS One. 2022 Jan 18;17(1):e0260668. doi: 10.1371/journal.pone.0260668 (PMC8765630; doi:10.1371/journal.pone.0260668)
Supplement: S9 File — (DOCX) [file pone.0260668.s009.docx]

QUESTIONNAIRE #1

CONSUMERS

1. Personal datas; Please circle your answer!
   1. Nationality:...1-dagomba; 2-gonja; 3-mamprusi; 4-ewe; 5-hausa; 6-akan........
   2. Place of live in Tamale: ..................................................................................................
   3. Age
      1. 0-10 years
      2. 11-20 years
      3. 21-30 years
      4. 31-50
      5. 50+ years
   4. Gender
      1. Male
      2. Female
   5. Religion
      1. Islamic
      2. Christanity
      3. Hinduism
      4. Atheism
      5. Non public
      6. Other, please specify:............................................................................................
   6. Education
      1. Non formal
      2. Primary
      3. Secondary ( High-schools, Gymnasium, Technician school)
      4. Tertiary
   7. How many children do you have?

.............

- 1. How many of them live with you?

.............

- 1. How many of you live in the household?

.............

1. How many times did you eat in the past one week?

|  | **Times per week** | | | | |
| --- | --- | --- | --- | --- | --- |
| **Breakfast** | 0 | 1x | 2-4x | >4x | NA |
| **Lunch** | 0 | 1x | 2-4x | >4x | NA |
| **Supper** | 0 | 1x | 2-4x | >4x | NA |
| **Snack** | 0 | 1x | 2-4x | >4x | NA |
| _______ | 0 | 1x | 2-4x | >4x | NA |

1. How many times did you eat meat in the past week?

|  | **Times per week** | | | | |
| --- | --- | --- | --- | --- | --- |
| **Breakfast** | 0 | 1x | 2-4x | >4x | NA |
| **Lunch** | 0 | 1x | 2-4x | >4x | NA |
| **Supper** | 0 | 1x | 2-4x | >4x | NA |
| **Snack** | 0 | 1x | 2-4x | >4x | NA |
| _______ | 0 | 1x | 2-4x | >4x | NA |

1. How many times did you eat beef ( meat, intestines, sausages...) in the last week?

|  | **Times per week** | | | | |
| --- | --- | --- | --- | --- | --- |
| **Breakfast** | 0 | 1x | 2-4x | >4x | NA |
| **Lunch** | 0 | 1x | 2-4x | >4x | NA |
| **Supper** | 0 | 1x | 2-4x | >4x | NA |
| **Snack** | 0 | 1x | 2-4x | >4x | NA |
| _______ | 0 | 1x | 2-4x | >4x | NA |

1. How many times did you eat chicken ( meat, sausages...) in the last week?

|  | **Times per week** | | | | |
| --- | --- | --- | --- | --- | --- |
| **Breakfast** | 0 | 1x | 2-4x | >4x | NA |
| **Lunch** | 0 | 1x | 2-4x | >4x | NA |
| **Supper** | 0 | 1x | 2-4x | >4x | NA |
| **Snack** | 0 | 1x | 2-4x | >4x | NA |
| _______ | 0 | 1x | 2-4x | >4x | NA |

1. How many times did you eat fish (fried, smoked, can ...) in the last week?

|  | **Times per week** | | | | |
| --- | --- | --- | --- | --- | --- |
| **Breakfast** | 0 | 1x | 2-4x | >4x | NA |
| **Lunch** | 0 | 1x | 2-4x | >4x | NA |
| **Supper** | 0 | 1x | 2-4x | >4x | NA |
| **Snack** | 0 | 1x | 2-4x | >4x | NA |
| _______ | 0 | 1x | 2-4x | >4x | NA |

1. How many times did you eat pork ( meat, frankfurites) in the last week?

|  | **Times per week** | | | | |
| --- | --- | --- | --- | --- | --- |
| **Breakfast** | 0 | 1x | 2-4x | >4x | NA |
| **Lunch** | 0 | 1x | 2-4x | >4x | NA |
| **Supper** | 0 | 1x | 2-4x | >4x | NA |
| **Snack** | 0 | 1x | 2-4x | >4x | NA |
| _______ | 0 | 1x | 2-4x | >4x | NA |

1. How many times did you eat home prepared food in the past week?

|  | **Times per week** | | | | |
| --- | --- | --- | --- | --- | --- |
| **Breakfast** | 0 | 1x | 2-4x | >4x | NA |
| **Lunch** | 0 | 1x | 2-4x | >4x | NA |
| **Supper** | 0 | 1x | 2-4x | >4x | NA |
| **Snack** | 0 | 1x | 2-4x | >4x | NA |
| _______ | 0 | 1x | 2-4x | >4x | NA |

1. Do you cook?
   1. Yes
   2. No
2. How many times did you cook in the past one week?

|  | **Times per week** | | | | |
| --- | --- | --- | --- | --- | --- |
| **Breakfast** | 0 | 1x | 2-4x | >4x | NA |
| **Lunch** | 0 | 1x | 2-4x | >4x | NA |
| **Supper** | 0 | 1x | 2-4x | >4x | NA |
| **Snack** | 0 | 1x | 2-4x | >4x | NA |
| _______ | 0 | 1x | 2-4x | >4x | NA |

1. How many times did you cook meat in the past one week?

|  | **Times per week** | | | | |
| --- | --- | --- | --- | --- | --- |
| **Breakfast** | 0 | 1x | 2-4x | >4x | NA |
| **Lunch** | 0 | 1x | 2-4x | >4x | NA |
| **Supper** | 0 | 1x | 2-4x | >4x | NA |
| **Snack** | 0 | 1x | 2-4x | >4x | NA |
| _______ | 0 | 1x | 2-4x | >4x | NA |

1. What type of finished meat products have you ever tried? ( in Ghana)
   1. Beef sausages
   2. Beef pepperoni
   3. Beef hamburger meat
   4. Chicken sausages
   5. Chicken breakfast sausages
   6. Frankfurties
   7. Bacon
   8. Other, please specify:.............................................................................................
2. If you have favourite, which is it?

.......................................................................................................................................................

1. How many times did you eat finished meat products (sausages, hamburger meat, frankfurties...) in the past one week?

|  | **Times per week** | | | | |
| --- | --- | --- | --- | --- | --- |
| **Breakfast** | 0 | 1x | 2-4x | >4x | NA |
| **Lunch** | 0 | 1x | 2-4x | >4x | NA |
| **Supper** | 0 | 1x | 2-4x | >4x | NA |
| **Snack** | 0 | 1x | 2-4x | >4x | NA |
| _______ | 0 | 1x | 2-4x | >4x | NA |

1. Where do you buy meat? Indicate your answer with ’X’!

|  | **Cold store, Supermarket** | **Roadside, Market butchers** | **Producing  factory** | **Self slaughter** | **Farmer** |
| --- | --- | --- | --- | --- | --- |
| **Beef** |  |  |  |  |  |
| **Chicken** |  |  |  |  |  |
| **Guinea Fowl** |  |  |  |  |  |
| **Pork** |  |  |  |  |  |
| **Mutton** |  |  |  |  |  |
| **Chevon** |  |  |  |  |  |
| **Red Fish** |  |  |  |  |  |
| **Brama** |  |  |  |  |  |
| **Mackerel** |  |  |  |  |  |
| **________** |  |  |  |  |  |
| **________** |  |  |  |  |  |

1. Do you pay attention to the origin of product?
   1. Yes, I always know where it is from
   2. Yes, I usually know where it is from
   3. No, I do not know the origin
2. What do you think of the origin of the following food-items? Depend on your consumption! ( If it is from abroad, please add the country or the continent)

|  | **Carcass,  bone-in/boneless meat** | **Finished-product  ( sausages, hamburger-meat, etc.)** |
| --- | --- | --- |
| **Beef** | Ghana/Abroad: | Ghana/Abroad: |
| **Chicken** | Ghana/Abroad: | Ghana/Abroad: |
| **Guinea fowl** | Ghana/Abroad: | Ghana/Abroad: |
| **Pork** | Ghana/Abroad: | Ghana/Abroad: |
| **Mutton** | Ghana/Abroad: | Ghana/Abroad: |
| **Chevon** | Ghana/Abroad: | Ghana/Abroad: |
| **Red Fish** | Ghana/Abroad: | Ghana/Abroad: |
| **Brama** | Ghana/Abroad: | Ghana/Abroad: |
| **Mackerel** | Ghana/Abroad: | Ghana/Abroad: |
| ________ | Ghana/Abroad: | Ghana/Abroad: |
| ________ | Ghana/Abroad: | Ghana/Abroad: |

1. Please compare local products to non-local products!

A: Agree strongly B: Agree little C: Disagree little D: Disagree strongly

- 1. Local meat tastes good A B C D
  2. Local meat is cheap A B C D
  3. Local meat is hygenic A B C D
  4. Variety of local products does satisfy my needs A B C D
  5. Local meat tastes bad A B C D
  6. Local meat is expensive A B C D
  7. Local meat is unhygenic A B C D
  8. Variety of local products does not satisfy my needs A B C D

1. Which of the following reason(s) is/are responsible for your preferance?
   1. Income
   2. Price of product
   3. Religion policies
   4. Taste
   5. Time to prepare
   6. Proximity to market
2. Which kind of meat do you buy?

|  | **Frozen** | **Chilled** | **Room temperature** |
| --- | --- | --- | --- |
| **Beef** |  |  |  |
| **Chicken** |  |  |  |
| **Guinea Fowl** |  |  |  |
| **Pork** |  |  |  |
| **Mutton** |  |  |  |
| **Chevon** |  |  |  |
| **Red Fish** |  |  |  |
| **Brama** |  |  |  |
| **Mackerel** |  |  |  |
| **________** |  |  |  |
| **________** |  |  |  |

1. Do you prepare the meat in the day you have bought it?
   1. Yes, everytime
   2. Yes, usually
   3. No, usually not
   4. No, never
2. If you do not, where do you store it?
   1. Fridge
   2. Freezer
   3. Kitchen
   4. Garden
   5. Other, please specify: ............................................................................................
3. **Your monthly income in Ghana Cedis (optional):**
   - 1. 0-200
     2. 201-800
     3. 801-1500
     4. 1501-3000
     5. 3000+
